# Distress, problems, referral wish, and supportive health care use in breast cancer survivors beyond the first year after chemotherapy completion

**DOI:** 10.1007/s00520-019-05030-6

**Published:** 2019-09-11

**Authors:** J. M. Admiraal, J. E. H. M. Hoekstra-Weebers, C. P. Schröder, W. Tuinier, G. A. P. Hospers, Anna K. L. Reyners

**Affiliations:** 1 University of Groningen, University Medical Center Groningen, Department of Medical Oncology, Groningen, The Netherlands; 2 University of Groningen, University Medical Center Groningen, Wenckebach Institute, Groningen, The Netherlands

**Keywords:** Breast cancer survivor, Distress, Distress Thermometer, Referral, Longitudinal

## Abstract

**Purpose:**

We examined distress levels, problems, referral wish, and supportive health care use in a cross-sectional group of breast cancer survivors at two-time points with a 1-year time interval. Also, factors related to continuing elevated distress were explored.

**Methods:**

Breast cancer survivors, 1–5 years after chemotherapy completion, filled in the Dutch Distress Thermometer/Problem List (DT/PL) and questions on background characteristics at study inclusion (T1). DT/PL responses and health care use were discussed during semi-structured interviews. One year later, re-assessment took place (T2). The data were analyzed by descriptive and univariate analyses. Continuing elevated distress was defined as a DT score ≥ 5 at T1 and T2.

**Results:**

Seventy-three survivors completed all questionnaires (response = 84.6%). Eighteen (25%) experienced continuing elevated distress. Fatigue (T1 *N* = 48 (66%); T2 *N* = 41 (56%)) and lack of physical fitness (T1 *N* = 44 (60%); T2 *N* = 36 (49%)) were most often reported. Time since diagnosis, health care use, and practical, social, emotional and physical problems were significantly associated with continuing elevated distress. Between diagnosis and T1, *N* = 49(67%) used supportive healthcare services, mostly a psychologist and/or a physical/lymphedema therapist, and between T1 and T2, 39 (53%) did. At T1, 8 (11%) expressed a referral wish and at T2, 11 (16%) did.

**Conclusions:**

Screening and management of distress, problems, and referral wish are important, even years after chemotherapy completion as a substantial proportion of breast cancer survivors continue to report elevated distress and problems. Special attention should be paid to survivors reporting physical problems, especially fatigue and lack of physical fitness, since these problems are most strongly related to continuing elevated distress.

## Introduction

Breast cancer is the most prevalent cancer in women worldwide [1]. Due to earlier diagnosis and advances in treatment, the 5-year survival rate of breast cancer increased from 74 to 88% between 1981 and 2015 in the Netherlands [2]. With the growing number of breast cancer survivors, understanding of not only physical but also psychosocial functioning beyond diagnosis and treatment is essential for optimal survivorship care for this population.

Receiving a diagnosis of cancer and undergoing treatment pose challenges to survivors’ coping abilities. Survivors can experience tumor- or treatment-related physical difficulties such as fatigue, insomnia, and sexuality-related problems, and these problems may persist into longer-term survivorship [3–6]. Additionally, they may face emotional, social, spiritual, and/or practical problems [7–9].

The problems patients experience altogether are often referred to as “distress” [10]. According to the transactional model of stress and coping of Lazarus and Folkman (1984), distress arises when an individual appraises that the demands of the stressor (e.g., cancer) exceed personal resources (the survivor’s ability to cope with cancer) [11]. Distress has been associated with reduced health-related quality of life [12, 13], low satisfaction with medical care [14], and decreased treatment adherence [15]. Distress seems transient for most cancer survivors, but 15–21% of the survivors report stable high levels of distress up to 15-month post-diagnosis [16, 17]. Consequently, it has been recommended to routinely screen for distress in cancer survivors to detect problems for which referral may be indicated. The National Comprehensive Cancer Network (NCCN, US) guideline for distress management was the first to recommend to screen all cancer patients with the Distress Thermometer (DT), a questionnaire specifically developed for cancer patients [10]. The NCCN also advises to use a Problem List (PL) that investigates which problem(s) in the practical, social, emotional, spiritual, and/or physical domain underlie the reported level of distress. The DT in combination with the PL has a good reliability and internal consistency [18]. A meta-analysis including 42 studies from 20 countries, including 14,808 cancer patients varying in cancer and treatment type, showed that the DT is a highly useful and valid screening tool to detect distress [19].

The Dutch guideline on screening and monitoring distress describes a process that encompasses (1) completion of the Dutch version of the DT/PL, including a question on referral wish, (2) discussion of the completed DT/PL with patients, and (3) referral to appropriate healthcare services if needed or wished [20]. Following the stress-coping model of Lazarus and Folkman, survivors should be approached on how to optimize their ability to cope and reduce their burden. The Dutch guideline describes a process that is in line with this stress-coping approach. Adequate referral to supportive health care services after exploring survivors’ referral wish can aid in enhancing effective coping strategies and reducing the burden of the problems experienced [20]. Also, a screening process coupled with discussion and referral according to pre-determined pathways showed to be more effective with respect to doctor-patient communication and number of referrals than a screening process without these components [21–23].

Although the DT/PL has been recommended as the preferred tool for screening [24], little is known about DT/PL responses of breast cancer survivors who are beyond the first year after completion of primary treatment, i.e., longer-term cancer survivors. Samples often include a mixture of survivors who are within the first year after primary treatment (re-entry phase [6]), long-term survivors (5+ years after diagnosis [25]), and survivors who are in between (longer-term survivors). This makes it difficult to gain insight into the severity of distress, nature of problems, and referral wish of longer-term survivors [26–28]. Additionally, several studies examined the course of distress over time in breast cancer survivors who were within the first 15 months after diagnosis [17, 29]. However, no studies are known assessing DT/PL responses over time in longer-term (breast) cancer survivors. As longer-term breast cancer survivors may suffer from lingering emotional and physical problems (e.g., [3]), longitudinal studies may provide important information about the prevalence of distress and the underlying problems over time. Also, knowledge about supportive health care use and referral wish can further increase insight into their needs.

The present study aims to contribute to our understanding of (clinically elevated) distress levels, problems, referral wish and health care use in longer-term breast cancer survivors over a 1-year time period. Moreover, we explored which sociodemographic and illness-related variables and underlying problems were associated with continuing elevated distress.

## Methods

### Patients

Survivors were recruited from the outpatient clinic of the Department of Medical Oncology of the University Medical Center Groningen (UMCG). Female breast cancer survivors who consecutively visited their medical oncologist for a routine follow-up visit and who had completed adjuvant chemotherapy 1–5 years earlier were invited to participate (longer-term survivors). Eligibility criteria were the following: age ≥ 18 years, stage I–III breast cancer, no recurrent cancer, physically and cognitively able to complete a questionnaire and be interviewed, and sufficiently fluent in Dutch. The study was approved by the medical ethical committee of the UMCG.

### Procedures

Eligible survivors received a letter at home with information about study aims and procedures, the questionnaire, an informed consent form, and contact information of the investigators, 1 week before the routine follow-up visit. Survivors deciding to participate were requested to return the completed informed consent form and questionnaire in a prepaid return envelope to the UMCG before the visit (T1). Informed consent was obtained from all individual survivors included in the study. Immediately after the routine follow-up visit, enrolled survivors received a semi-structured interview (± 20 min) with a specially trained oncology nurse or research psychologist in which responses on the DT/PL were discussed; brief psycho-education was provided (in case of problems); the need for a referral to additional supportive care services was explored and supportive health care use since diagnosis was assessed (first cross-sectional assessment; T1). Single sessions (e.g., intake) with a health care professional were not considered as care. Survivors expressing a referral wish were referred to a relevant health care professional or were instructed how to access the health care service that was requested.

The DT/PL was sent to participating survivors 1 year later together with an invitation for a second interview (second cross-sectional assessment; T2). Survivors who forgot to return the DT/PL or who were not scheduled for a follow-up visit (to a medical, surgical, or radiation oncologist) 1 year later were offered an interview by telephone. Up to three attempts by phone were made to contact survivors who did not return the DT/PL.

### Measures

Self-report questions assessed the following socio-demographic characteristics at T1: age, marital status, presence of children, educational level (range: primary (1) to university (6)), and employment status (employed for wages; not-employed). Illness-related characteristics (date of diagnosis, pTNM-classification, cancer stage (I–III, derived from pTNM-classification), medical treatment, and date of completion of last chemotherapy cycle were collected from the survivors’ medical records.

Distress, problems, and referral wish were measured using the Dutch DT/PL [8, 10], at T1 and T2. The DT consists of a single item that asks cancer survivors to indicate the amount of overall distress experienced during the past week on an 11-point scale (0–10; no to extreme distress). The Dutch DT/PL has been validated for cancer patients with different diagnoses and treatments [8, 9]. A DT cutoff score of ≥ 5 represents clinically elevated distress in Dutch breast cancer survivors. The sensitivity was 0.85, specificity 0.66, positive predictive value 0.32, and negative predictive value 0.96 [9]. On the 47-item PL, cancer survivors can indicate whether or not (yes/no) they experienced practical, family/social, emotional, religious/spiritual, and physical problems. Survivors were asked to rate from 1 to 10 the amount of distress they experienced for each item in the PL they answered “yes.” Internal consistency and reliability of the PL is good (Cronbach’s alpha = 0.90). Lastly, the questionnaire assesses cancer survivors’ referral wish (yes, maybe, or no) to a health care professional (psychologist, social or pastoral worker, oncology nurse, physical therapist, or dietician), peer support from a fellow patient, and/or to other types of health care [8].

During the interview at T1 and T2, patients were asked whether they had received care from a psychologist, social or pastoral worker, sexologist, physical therapist, lymphedema therapist, and dietician and/or whether they were enrolled in a rehabilitation program combining physical and cognitive-behavioral therapy. Uptake of other types of health care was also explored.

### Data analysis

The percentage of missing values ranged from 0 to 11.7%. Missing data patterns were examined with Little’s missing completely at random test with a chi-square statistic (*p* < 0.05), and descriptive analyses, i.e., separate-variance *t* tests, cross tabulations, and a tabulated pattern table. The results showed that the data could be assumed to be missing at random, i.e., missingness was predicted by variables that were part of the dataset. Five imputations were generated for the missing data by the use of the fully conditional specification algorithm (non-monotonous data) [30, 31]. The variables, referral wish, and referral by the research team were not imputed because of the small *N* for these variables and many possible outcomes: imputations were perceived as unreliable.

Descriptive statistics were calculated for sociodemographic and illness-related characteristics, the DT/PL, and health care use. Continuing elevated distress was defined as a DT score of ≥ 5 at both T1 and T2. Continuing low distress was assumed when patients reported a DT score of < 5 at both timepoints. Survivors who developed recurrent/metastasized breast cancer or another malignancy during the study period were excluded at T2.

Associations between DT/PL scores and changes herein over time (ΔT1–T2) and time since diagnosis and time since chemotherapy were calculated by Spearman’s correlations to explore the effect of time on DT/PL responses. Chi-square tests and Mann-Whitney *U* tests were performed to explore univariate effects of sociodemographic and illness-related characteristics, health care use (dichotomized (Yes/No), problem domains and problem items at T1 on continuing elevated distress (patients with a DT score of ≥ 5 at both T1 and T2 versus patients with a DT score of ≤ 4 at one or both time points). Effect sizes (ES) were reported for significant results to examine the clinical relevance (Cramers’s V (V) for chi-square tests; *r* for Mann-Whitney *U* tests). An ES of 0.1 indicates a small effect, 0.3 a medium effect, and 0.5 a large effect [32, 33]. Statistical analyses were performed using SPSS, version 24 (SPSS Inc. Chicago, IL).

## Results

### Patient characteristics

**Fig. 1:**
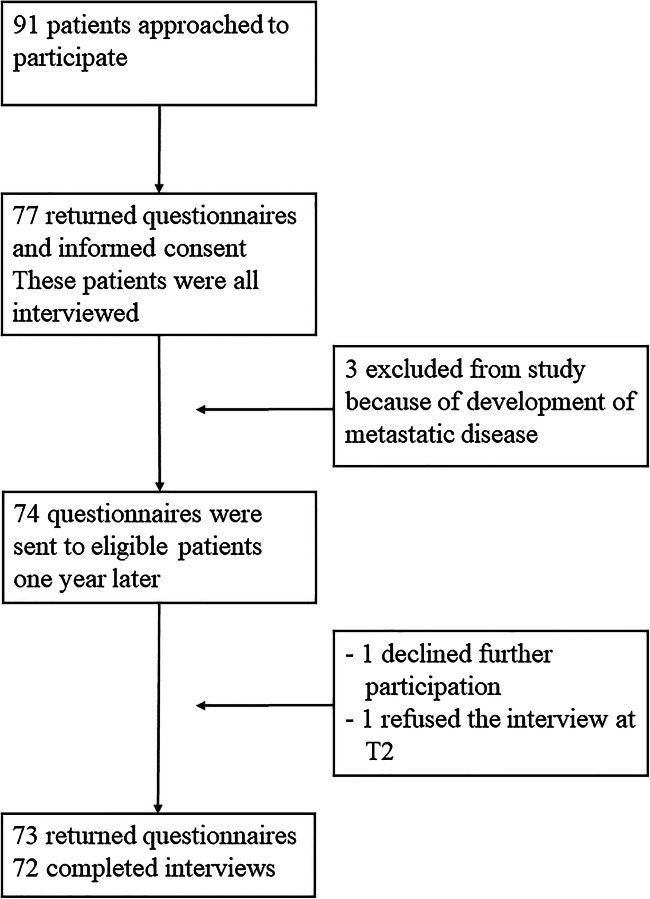
Flow chart of the study

Seventy-seven of the 91 eligible survivors approached during the 1.5 year of the study at the UMCG and agreed to participate in the study (response = 84.6%; Fig. 1). The main reasons for survivors to decline study participation were a lack of interest to talk about their current psychosocial health status (*N* = 7) or they did not feel comfortable discussing psychosocial issues (*N* = 3). Chemotherapy completion varied between 1.0 and 4.8 years at study entry, and patients were on average 3 years after diagnosis (range = 1.6–5.2). Fifty-four (74%) patients were receiving hormonal treatment at T1; 44 at T2 (60%, Table 1).

**Table 1:** Patient characteristics at T1 and univariate associations with continuing elevated distress (*N* = 73)

Characteristic	Median (IQR)	*N* (%)	Test statistic^ab^ (*p*)
Age (years)	52.1 (45.7–56.6)		*U* = 437.8 (0.440)
Marital status			
Single/widowed/divorced		12 (16.4)	*χ*^*2*^ = 0.0 (0.967)
Married/living together		61 (83.6)	
Children			
No		21 (28.8)	
Yes		52 (71.2)	*χ*^*2*^ = 1.1 (0.292)
Educational level	4.0 (3.0–5.0)		*U* = 491.5 (0.928)
Employment			
Not employed		30 (41.1)	*χ*^*2*^ = 3.8 (0.054)
Employed		43 (58.9)	
Stage of cancer			
I		11 (15.1)	χ^2^ = 1.4 (0.495)
II		49 (67.1)	
III		13 (17.8)	
Time since diagnosis (years)	2.8 (2.1–3.7)		*U* = 336.2 (0.040)
Time since chemotherapy completion (years)	2.2 (1.6–3.4)		*U* = 397.0 (0.198)
Surgery			
Lumpectomy		31 (42.5)	*χ*^*2*^ = 0.7 (0.406)
Mastectomy		42 (57.5)	
Systemic adjuvant therapy			
Chemotherapy		12 (16.4)	*χ*^*2*^ = 6.5 (0.091)
Chemotherapy and immunotherapy		7 (9.6)	
Chemotherapy and hormonal therapy		45 (61.6)	
Chemotherapy, immunotherapy, and hormonal therapy		9 (12.3)	
Hormonal therapy at T1			
No/completed		19 (26.0)	*χ*^*2*^ = 0.0 (0.871)
Yes		54 (74.0)	
Hormonal therapy at T2			
No/completed^a^		29 (39.7)	*χ*^*2*^ = 0.0 (0.899)
Yes		44 (60.3)	
Adjuvant radiotherapy			
No		31 (42.5)	*χ*^*2*^ = 0.5 (0.488)
Yes		42 (57.5)	
Breast reconstruction			
Between diagnosis and T1			
No		57 (78.1)	*χ*^*2*^ = 0.4 (0.509)
Yes		16 (21.9)	
Between T1 and T2			
No		59 (80.8)	*χ*^*2*^ = 0.1 (0.726)
Yes		14 (19.2)	
Health care use			
Between diagnosis and T1			
No		24 (32.9)	*χ*^*2*^ = 8.2 (0.004)
Yes		49 (67.1)	
Between T1 and T2			
No		34 (46.6)	*χ*^*2*^ = 5.5 (0.022)
Yes		39 (53.4)	

^a^Average test result across imputed datasets

^b^Continuing elevated distress was defined as a DT score of ≥ 5 at both T1 and T2

*U*, Mann-Whitney *U* test; *X*^2^, chi-square; *IQR*, interquartile range

### Distress and underlying problems

At T1, 35 survivors (48%) indicated they experienced clinically elevated distress. Twenty-three (32%) reported elevated distress at T2. Eighteen survivors (25%) suffered from clinically elevated levels of distress both at T1 and 1 year later. Thirty-three (46%) reported low distress at both timepoints (Table 2). The top five most often reported problems were in the physical and emotional domains (Table 3). Fatigue and lack of physical fitness were most often reported at T1 and T2, both by the complete sample and by survivors experiencing continuing elevated distress. Problems were more prevalent in the continuing elevated distress subgroup (e.g., fatigue: *N* = 16/18; 89% (T1)) compared to the complete sample (fatigue: *N* = 48/73; 66% (T1)). The number of survivors who reported one of the top five problems decreased from T1 to T2. At T2, more survivors with continuing elevated distress reported tension/nervousness and fears than at T1.

**Table 2:** Descriptives of the DT/PL scores at study entry (T1) and 1 year later (T2), changes over time, and univariate associations between the problem domains and continuing elevated distress

	T1		T2		Continuing elevated distress
	*N* (%)	Median (IQR)	*N* (%)	Median (IQR)	Test statistic (*p*)^a^
DT		4.0 (1.5–6.0)		3.0 (1.0–5.0)	
*N* scoring ≥ 5	35 (48)		23 (32)		
Elevated distress at T1 and T2			18 (25)		
Low distress at T1 and elevated distress at T2			5 (7)		
Elevated distress at T1 and low distress at T2			17 (23)		
Low distress at T1 and T2			33 (45)		
PL subscales					
Practical (0–70)^r^		4.0 (0.0–11.5)		0.0 (0.0–8.0)	*U* = 255.4 (0.002)
Social (0–30)^r^		0.0 (0.0–5.0)		0.0 (0.0–1.7)	*U* = 371.7 (0.050)
Emotional (0–100)^r^		12.0 (3.0–35.0)		9.1 (0.0–22.0)	*U* = 279.7 (0.005)
Spiritual (0–20)^r^		0.0 (0.0–0.0)		0.0 (0.0–0.1)	*U* = 440.1 (.297)
Physical (0–250)^r^		9.5 (9.5–46.5)		7.5 (15.4–34.0)	*U* = 181.4 (0.000)

^r^Possible scoring range; ^a^Average test result across imputed datasets; *U*, Mann-Whitney *U* test; IQR, interquartile range

**Table 3:** Top 5 distress-related problems at study entry (T1) and 1 year later (T2) for the complete sample and for patients with continuing elevated distress

	T1				T2			
Rank	Complete sample	(%)	Distressed survivors^a^	(%)	Complete sample	(%)	Distressed survivors^a^	(%)
1	Fatigue	(66)	Fatigue	(89)	Fatigue	(56)	Lack of physical fitness	(83)
2	Lack of physical fitness	(60)	Lack of physical fitness	(89)	Lack of physical fitness	(49)	Fatigue	(78)
3	Tension/nervousness	(58)	Lack of muscle strength	(78)	Tension/nervousness	(48)	Tension/nervousness	(78)
4	Fears	(56)	Pain	(72)	Sleeping problems	(45)	Fears	(72)
5	Depression	(52)	Tension/nervousness	(67)	Fears	(44)	Sleeping problems	(67)
			Fears	(67)				
			Depression	(67)				
			Concentration problems	(67)				

^a^Survivors who experienced continuing elevated distress defined as a DT score of ≥ 5 at both T1 and T2

### Variables associated with continuing elevated distress versus no continuing elevated distress

Time since diagnosis and time since chemotherapy were not related to DT/PL scores at T1 or T2 and DT/PL change scores over time. Shorter time since diagnosis was significantly related to a higher likelihood of reporting clinically elevated distress at both measurement points, i.e., to experience continuing elevated distress (Table 1; *r* = 0.24; small to medium ES). Also, survivors who accessed health care services between diagnosis and T1 (*V* = 0.34; medium ES) and/or between T1 and T2 (*V* = 0.28; small to medium ES) were more likely to report continuing elevated distress (Table 1). Survivors who had a higher score on the practical (*V* = 0.38; medium to large ES), social (*V* = 0.23; small to medium ES), emotional (*V* = 0.33; medium ES), and/or physical (*V* = 0.47; large ES) problem domains at T1 were more likely to indicate that they experienced continuing elevated distress (Table 2).

### Referral wish, health care use, and actual referral to specific services

Table 4 displays survivors’ health care use, referral wish, actual referral at T1 and T2, and uptake of T1 referrals. At T1, 27 survivors considered (37%), and eight (11%) had a referral wish to a psychosocial and/or paramedical health care provider (Table 2). Of these last, two indicated they recently started receiving care for their needs. At T2, 12 considered (17%), and 11 wished (16%) a referral. Two of the eleven had recently been referred (Table 4).

**Table 4:** Supportive health care service use, referral wish, actual referral by the research team for T1 and T2, and uptake of T1 referrals (*N* = 73)

	Health care use between diagnosis and T1 (*N* (%))	Referral wish at T1 (*N* (%))^a^	Referred by research team at T1 (*N* (%))	Health care use between T1 and T2 (*N* (%))		Referral wish at T2 (*N* (%))^ab^	Referred by research team at T2 (*N* (%))^b^
No	24 (33)	38 (52)	63 (86)	34 (47)		48 (68)	65 (92)
Maybe		27 (37)				12 (17)	
Yes	49 (67)	8 (11)	10 (14)	39 (53)		11 (16)	6 (8)
From/to 1 provider	33 (45)	5 (7)	10 (14)	30 (41)		7 (10)	6 (8)
From/to 2 providers	12 (16)	2 (3)	0 (0)	7 (10)		0 (0)	0 (0)
From/to ≥ 3 providers	4 (6)	1 (1)	0 (0)	3 (4)		4 (6)	0 (0)
Health care type^c^				Continued	New uptake		
Additional medical or nursing care							
Breast cancer nurse/nurse specialist	0 (0)		0 (0)	0 (0)	0 (0)	0 (0)	0 (0)
Oncology/nurse practitioner		2 (3)				3 (4)	
Gynecologist/sexologist (total)	0 (0)	0 (0)	1 (1)	0 (0)	0 (0)	0 (0)	3 (4)
Actual uptake of T1 referral					0 out of 1	0 (0)	
Paramedical health care							
Physical therapist/lymphedema therapist	18 (*2*5)	0 (0)	0 (0)	5 (7)	20 (27)	1 (1)	0 (0)
Dietician	0 (0)	2 (3)	0 (0)	0 (0)	3 (4)	4 (6)	0 (0)
Psychosocial health care							
Psychologist (total)	27 (37)	7(10)	6 (8)	7 (10)	3 (4)	4 (6)	3 (4)
Actual uptake of T1 referral					3 out of 6		
Social worker (total)	7 (10)	1 (1)	2 (3)	1 (1)	0 (0)	3 (4)	0 (0)
Actual uptake of referral at T1					0 out of 2		
Psychiatrist	4 (6)	0 (0)	0 (0)	3 (4)	2 (3)	0 (0)	0 (0)
Pastoral worker	1 (1)	0 (0)	0 (0)	1 (1)	0 (0)	0 (0)	0 (0)
Other supportive care services							
Multidimensional rehabilitation program (total)	11 (15)	0 (0)	1 (1)	0 (0)	4 (6)	0 (0)	0 (0)
Actual uptake of T1 referral					1 out of 1		
Other type of (supportive) health care^c^	2 (3)	0 (0)	0 (0)	1 (1)	3 (4)	3 (4)	0 (0)

^a^Survivors who answered “No” and “Maybe” on the referral wish question are taken together

^b^Answers from 2 survivors on the referral by the research team question are missing; thus *N* = 71 at T2

^c^Both inside and outside the University Medical Center Groningen (UMCG)

^d^Other types of health care include general practitioner, support from fellow patients, and creative therapy

Between diagnosis and T1, supportive care was most often received from a psychologist (*N* = 27; 37%) or a physical therapist/lymphedema therapist (*N* = 18; 25%). The most frequently visited health care providers between T1 and T2 were the physical/lymphedema therapist (*N* = 25; 34%)) and the psychologist (*N* = 10; 14%). At T1, ten survivors (14%) were referred by the research nurse/psychologist after discussion of DT/PL responses, most (*N* = 6) to a psychologist. At T2, 11 (16%) survivors indicated having a referral wish and six (8% of the total group) were referred after discussion of DT/PL responses, three to a gynecologist/sexologist, and three to a psychologist. The one survivor who had been referred to a multidimensional rehabilitation program at T1 actually participated in the program. Of the six survivors referred to a psychologist, three actually went. The two survivors referred to a social worker and the one referred to a gynecologist/sexologist did not uptake the care service.

## Discussion

This study was the first that longitudinally assessed the DT/PL in breast cancer survivors who were 1 to 5 years after chemotherapy completion and 19 months to 5 years after diagnosis. The findings of the present study are that at least one-third of long-term survivors experienced clinically elevated distress at one of the time points, and one in four survivors reported clinically elevated distress at both time points. This proportion lies well above estimates of psychological morbidity in the general Dutch female population (between 10 and 19%; [34]). The percentage of patients with continuing elevated distress was somewhat higher than the 21% that was reported in a recent Dutch study that used the Dutch DT/PL and included survivors who were at 15-month post-diagnosis [17]. This difference may be explained by the treatment modalities survivors received: 63% of survivors in the aforementioned study received radiotherapy only whereas all survivors in the current study received chemotherapy. Patients who (also) receive chemotherapy in contrast to patients who receive other treatment modalities report higher levels of distress [29, 35] and lower emotional functioning at least up to 2 years after diagnosis [36]. The current study shows that a substantial proportion of breast cancer survivors experience clinically elevated levels of distress, even beyond the first year after chemotherapy completion.

Fatigue and lack of physical fitness were the most often reported problems as was reported previously [17, 28]. Also, the finding that emotional problems such as tension/nervousness and fears were frequently reported was in line with previous research. However, the prevalence of these problems was high compared to previous studies [17, 28], especially for emotional problems: other studies with the DT/PL reported percentages of 40% or lower [17, 28]. This may again be explained by the fact that all survivors in the current study received chemotherapy. Also, the women in our study were somewhat younger (mean = 50.6; median = 52.1) compared to the other studies (mean = 57 [28]; median = 58 [17]) which may explain the higher distress levels [9]. The number of survivors who reported physical and emotional problems decreased over the 1-year period but the numbers remained substantial. Also, a higher score for these problems at study inclusion, especially physical problems, increased the likelihood that survivors reported continuing elevated distress. Thus, problems which underlie distress can endure for years after chemotherapy completion in breast cancer survivors. Attention of health care professionals to lingering problems remains important, even years after chemotherapy as these problems and distress may not resolve without additional support.

Our findings show that a large proportion of survivors was or had been using supportive health care services. Notably, the use of a physical/lymphedema therapist increased from 25 to 36% over time. First symptoms of arm lymphedema may occur up to 3 years after surgery [37, 38] and can explain the increase in use of this service. Also, specific problems such as a lack of physical fitness and a lack of muscle strength, which are highly prevalent among survivors in the current study, may have encouraged survivors to seek help from a physical therapist. More than a third of survivors indicated they had received care from a psychologist before they participated in the study. A study including all types of German cancer patients reported that approximately 30% had used psychotherapy and/or psychological counseling since they got cancer. The authors showed that women and patients with symptoms of depression and anxiety were more likely to use these services [39].

The referral wishes survivors reported and consequent referral made by a member of the research team after discussion of the DT/PL did not always align. This could in part be explained by the fact that some survivors recently started receiving care for their needs through self-referral. More importantly, the discussion of DT/PL responses helped survivors to elucidate which problems were most troubling and what type of support was (mostly) needed. Consequently, the wish to be referred to a professional of a certain discipline changed. This result underlines the importance of discussing DT/PL responses as is being recommended in the Dutch guideline on distress management [20].

Our study had a number of limitations. The first is the small sample size. Therefore, we performed univariate analyses to explore associates of continuing elevated distress. Larger samples in future studies are needed to confirm and extend the current results. Second, a large variation existed between the time of chemotherapy completion and the first measurement (between 1 and 5 years after completion). However, the survivors seem to be a relatively homogeneous group considering the problems and health care use that were reported at both time points. Future research measuring survivors at fixed time-points during the illness trajectory can more precisely indicate survivors’ distress, problems, and health care use at specific time-points after chemotherapy completion. Finally, due to our study design, we were not able to assess whether survivors identified with continuing elevated distress experienced these levels of distress during the entire 1-year study period or at the measurement points only nor whether they suffered from elevated distress (at any time) during chemotherapy treatment.

Several important strengths can be noted. The current study was the first to longitudinally examine DT/PL responses in longer-term (breast) cancer survivors, particularly in those who were treated with chemotherapy as they often experience more problems than survivors who received other treatment types [9]. Second, we explored what problem domains were related to continuing elevated distress. One previous study related problem domains to distress but had a cross-sectional design and was performed in lung cancer patients [40]. Third, to the best of our knowledge, this is the first study that made an attempt to explore referral wish as measured with the DT/PL in combination with actual referral and uptake.

Considering the substantial proportion of longer-term breast cancer survivors who report (continuing) elevated distress, distress-related problems, and referral wishes, distress screening and management remain important in clinical practice even years after completion of chemotherapy. Physical problems, especially fatigue and lack of physical fitness, were most prevalent and were most strongly related to reporting continuing elevated distress. We recommend to pay special attention to survivors experiencing these problems, i.e., to discuss the impact of these problems on survivors’ lives, to inform survivors about the potential health risk of not treating these problems, and to discuss possible (self-management) strategies for dealing with these distress-underlying problems.

## References

[1] Jemal A, Bray F, Center MM, Ferlay J, Ward E, Forman D (2011). Global cancer statistics. CA Cancer J Clin.

[2] The Netherlands Cancer Registry (2011-2019) Dutch cancer figures retrieved from http://www.cijfersoverkanker.nl/selecties/overleving_borst/img568bab4dec98c?language=en. Accessed 6 Dec 2018

[3] Arndt V, Merx H, Stegmaier C, Ziegler H, Brenner H (2005). Persistence of restrictions in quality of life from the first to the third year after diagnosis in women with breast cancer. J Clin Oncol.

[4] Hartl K, Engel J, Herschbach P, Reinecker H, Sommer H, Friese K (2010). Personality traits and psychosocial stress: quality of life over 2 years following breast cancer diagnosis and psychological impact factors. Psychooncology.

[5] Knobf MT (2011). Clinical update: psychosocial responses in breast cancer survivors. Semin Oncol Nurs.

[6] Stanton AL (2012). What happens now? Psychosocial care for cancer survivors after medical treatment completion. J Clin Oncol.

[7] Carlson LE, Bultz BD (2003). Cancer distress screening. Needs, models, and methods. J Psychosom Res.

[8] Tuinman MA, Gazendam-Donofrio SM, Hoekstra-Weebers JE (2008). Screening and referral for psychosocial distress in oncologic practice: use of the distress thermometer. Cancer.

[9] Admiraal JM, Reyners AK, Hoekstra-Weebers JE (2013). Do cancer and treatment type affect distress?. Psychooncology.

[10] National Comprehensive Cancer Network (2014) NCCN Clinical practice guidelines in oncology: distress management V1. http://www.nccn.org/professionals/physician_gls/f_guidelines.asp#supportive. Accessed 6 Dec 2018 10.6004/jnccn.2019.0048 PMC6907687 31590149

[11] Shim EJ, Mehnert A, Koyama A, Cho SJ, Inui H, Paik NS, Koch U (2006). Health-related quality of life in breast cancer: a cross-cultural survey of German, Japanese, and South Korean patients. Breast Cancer Res Treat.

[12] Pelletier G, Verhoef MJ, Khatri N, Hagen N (2002). Quality of life in brain tumor patients: the relative contributions of depression, fatigue, emotional distress, and existential issues. J Neuro-Oncol.

[13] Von Essen L, Larsson G, Oberg K, Sjoden PO (2002). ‘Satisfaction with care’: associations with health-related quality of life and psychosocial function among Swedish patients with endocrine gastrointestinal tumours. Eur J Cancer Care (Engl).

[14] Kennard BD, Stewart S, Olvera R, Bawdon RE, Hailin AE, Lewis CP, Winick NJ (2004). Nonadherence in adolescent oncology patients: preliminary data on psychological risk factors and relationships to outcome. J Clin Psychol Med Settings.

[15] Henselmans I, Helgeson VS, Seltman H, de Vries J, Sanderman R, Ranchor AV (2010). Identification and prediction of distress trajectories in the first year after a breast cancer diagnosis. Health Psychol.

[16] Lo-Fo-Wong DN, de Haes HC, Aaronson NK, van Abbema DL, den Boer MD, van Hezewijk M, Immink M, Kaptein AA, Menke-Pluijmers MB, Reyners AK, Russell NS, Schriek M, Sijtsema S, van Tienhoven G, Sprangers MA (2016). Predictors of enduring clinical distress in women with breast cancer. Breast Cancer Res Treat.

[17] Hoffman BM, Zevon MA, D’Arrigo MC, Cecchini TB (2004). Screening for distress in cancer patients: the NCCN rapid-screening measure. Psychooncology.

[18] Ma X, Zhang J, Zhong W, Shu C, Wang F, Wen J, Zhou M, Sang Y, Jiang Y, Liu L (2014). The diagnostic role of a short screening tool--the distress thermometer: a meta-analysis. Support Care Cancer.

[19] Working Party Guideline Screening for Psychosocial Distress (2017) Dutch nation-wide guideline: screening for psychosocial distress, version 2.0. http://www.oncoline.nl/detecteren-behoefte-psychosociale-zorg. Accessed 8 Dec 2018

[20] Klinkhammer-Schalke M, Koller M, Steinger B, Ehret C, Ernst B, Wyatt JC, Hofstadter F, Lorenz W, Regensburg QoL Study Group (2012). Direct improvement of quality of life using a tailored quality of life diagnosis and therapy pathway: randomised trial in 200 women with breast cancer. Br J Cancer.

[21] Carlson LE, Groff SL, Maciejewski O, Bultz BD (2010). Screening for distress in lung and breast cancer outpatients: a randomized controlled trial. J Clin Oncol.

[22] Girgis A, Breen S, Stacey F, Lecathelinais C (2009). Impact of two supportive care interventions on anxiety, depression, quality of life, and unmet needs in patients with nonlocalized breast and colorectal cancers. J Clin Oncol.

[23] Donovan KA, Grassi L, McGinty HL, Jacobsen PB (2014). Validation of the distress thermometer worldwide: state of the science. Psychooncology.

[24] Bloom JR, Petersen DM, Kang SH (2007). Multi-dimensional quality of life among long-term (5+ years) adult cancer survivors. Psychooncology.

[25] Lester J, Crosthwaite K, Stout R, Jones RN, Holloman C, Shapiro C, Andersen BL (2015). Women with breast cancer: self-reported distress in early survivorship. Oncol Nurs Forum.

[26] McFarland DC, Shaffer KM, Tiersten A, Holland J (2018). Prevalence of physical problems detected by the distress thermometer and problem list in patients with breast cancer. Psychooncology.

[27] Ploos van Amstel FK, van den Berg SW, van Laarhoven HW, Gielissen MF, Prins JB, Ottevanger PB (2013). Distress screening remains important during follow-up after primary breast cancer treatment. Support Care Cancer.

[28] Bidstrup PE, Christensen J, Mertz BG, Rottmann N, Dalton SO, Johansen C (2015). Trajectories of distress, anxiety, and depression among women with breast cancer: looking beyond the mean. Acta Oncol.

[29] Garson GD (2015). Missing values analysis and data imputation.

[30] van Buuren S (2007). Multiple imputation of discrete and continuous data by fully conditional specification. Stat Methods Med Res.

[31] Field AP (2009). Discovering statistics using SPSS: (and sex and drugs and rock ‘n’ roll).

[32] Kim HY (2017). Statistical notes for clinical researchers: chi-squared test and Fisher’s exact test. Restor Dent Endod.

[33] Koeter MWJ, Ormel J (1991). Nederlandse bewerking en handleiding. [general health questionnaire: Dutch edition and manual].

[34] van Scheppingen C, Schroevers MJ, Smink A, van der Linden YM, Mul VE, Langendijk JA, Coyne JC, Sanderman R (2011). Does screening for distress efficiently uncover meetable unmet needs in cancer patients?. Psychooncology.

[35] Kootstra J, Hoekstra-Weebers JE, Rietman H, de Vries J, Baas P, Geertzen JH, Hoekstra HJ (2008). Quality of life after sentinel lymph node biopsy or axillary lymph node dissection in stage I/II breast cancer patients: a prospective longitudinal study. Ann Surg Oncol.

[36] Norman SA, Localio AR, Potashnik SL, Simoes Torpey HA, Kallan MJ, Weber AL, Miller LT, Demichele A, Solin LJ (2009). Lymphedema in breast cancer survivors: incidence, degree, time course, treatment, and symptoms. J Clin Oncol.

[37] Paskett ED, Naughton MJ, McCoy TP, Case LD, Abbott JM (2007). The epidemiology of arm and hand swelling in premenopausal breast cancer survivors. Cancer Epidemiol Biomark Prev.

[38] Faller H, Weis J, Koch U, Brahler E, Harter M, Keller M, Schulz H, Wegscheider K, Boehncke A, Hund B, Reuter K, Richard M, Sehner S, Wittchen HU, Mehnert A (2017). Utilization of professional psychological care in a large German sample of cancer patients. Psychooncology.

[39] Graves KD, Arnold SM, Love CL, Kirsh KL, Moore PG, Passik SD (2007). Distress screening in a multidisciplinary lung cancer clinic: prevalence and predictors of clinically significant distress. Lung Cancer.

